# Impact of chemical and herbal pre-treatments on shelf life and quality characteristics of dried mango (*Mangifera Indica* L.) slices

**DOI:** 10.1371/journal.pone.0360666

**Published:** 2026-10-13

**Authors:** Safeer Ahmad, Moazzam Rafiq Khan, Rana Muhammad Aadil, Beenish Israr

**Affiliations:** 1 National Institute of Food Science and Technology, University of Agriculture, Faisalabad, Pakistan; 2 Institute of Home Sciences, University of Agriculture, Faisalabad, Pakistan; Universidad Autonoma de Chihuahua, MEXICO

## Abstract

The present research was carried out to study and determine the effects of chemical and herbal pre-treatments on dried mango slices stored for 180 days under ambient conditions. Discoloration, antioxidants, loss of nutrients, off-flavour, and microbial growth during storage were reported to be concerning parameters. The parameters included in the study were proximate composition, physicochemical analysis, antioxidant analysis, microbial analysis, and color analysis. Score for vitamin C content, antioxidants, and color analysis, was better in pre-treated samples as compared to untreated dried mango samples, but showed a decreasing trend with an increase in storage interval. The analysis conducted at various intervals of storage depicted that the herbal pre-treatments preserved the vitamin C and antioxidant content, and comparable results were recorded. The microbial population in untreated dried mango slices was recorded as more than pre-treated, but was under the permissible limit. Overall, it was concluded that the use of herbal pre-treatments in comparison to chemicals was also effective for the preservation of nutritionally good quality dried fruit.

## Introduction

Mango (*Mangifera indica* L.) is a climacteric fruit widely cultivated in frost-free tropical and warm subtropical regions of the world. It belongs to the family Anacardiaceae and is believed to have originated in Southeast Asia [1]. Pakistan ranks as the fifth largest mango-producing country, with an annual production of approximately 1.77 million tonnes, contributing nearly 3.2% to the global mango export market and standing ninth among mango-exporting nations. The majority of mango production in Pakistan is concentrated in the Punjab province, followed by Sindh, with major mango-growing areas including Multan, Rahim Yar Khan, and Mirpur Khas [2]. Mango is commonly referred to as the “king of fruits” due to its appealing color, distinctive aroma and taste, and high nutritional value. Despite its economic and nutritional importance, mango is highly perishable and possesses a relatively short postharvest life, making it susceptible to significant losses. Postharvest losses of mango have been reported to range from 25% to 40% between harvesting and final consumption, primarily due to inadequate postharvest handling practices and unfavourable environmental conditions such as high temperatures [3].

Because mangos are a seasonal fruit and deteriorate quickly, adding value through processing and preservation is considered an efficient way to reduce these losses [4]. Mango processing helps stabilize prices, increase farmers’ income, and improve nutritional security at the societal level, in addition to reducing market glut during the busiest harvesting seasons. One of the most useful preservation methods for increasing fruit shelf life and lowering post-harvest losses is drying. However, long drying times or high temperatures are common in typical air-drying techniques, which may negatively impact product quality, qualities like color, flavor, texture, rehydration capability, and nutritional makeup. Heat-sensitive bioactive substances are extremely susceptible to thermal deterioration, especially carotenoids and vitamin C. To mitigate these quality losses, pre-treatments such as blanching and osmotic dehydration are frequently applied before drying to improve the physicochemical and nutritional characteristics of dried fruits [5]. Furthermore, the application of antibrowning chemicals to regulate both enzymatic and non-enzymatic browning reactions has been thoroughly researched. It has been documented that chemical preservatives such as potassium sorbate and potassium metabisulphite successfully prevent browning and quality degradation during processing and storage [6]. However, because of their possible negative consequences, the increasing prevalence of artificial chemicals and preservatives in processed foods has sparked health concerns. As a result, customers showed a growing inclination for less processed and organically preserved food items. Lemon juice has been identified as a natural preservative with antibacterial action, flavor enhancement, and color retention potential. Similarly, bioactive chemicals like gingerol, which have antibacterial activities, are responsible for the significant preservation qualities of ginger, a popular spice.

The application of suitable pre-treatments before drying plays a crucial role in accelerating moisture removal, preserving quality attributes, and reducing energy consumption. Pre-treatments may also inactivate deteriorative enzymes, thereby minimizing color changes and improving the overall acceptability of dried products [7]. Therefore, the present study was undertaken to evaluate the effects of selected chemical and herbal pre-treatments on the quality and storage stability of dried mango slices over a storage period of 180 days.

## Materials and methods

### Procurement of raw material

The early-season, commercially available mango cultivar ‘Yakta’ was obtained from the Mango Research Station, Shujaabad, Multan. All chemicals, reagents, and analytical standards employed in this study were procured from Sigma-Aldrich (St. Louis, MO, USA). The experimental work was carried out at the National Institute of Food Science and Technology, University of Agriculture, Faisalabad.

### Sample preparation

The drying procedures followed Ahmad et al.‘s (2024) description [8]. The mango fruit was washed with running water and then peeled and sliced into approximately 4–5 mm thicknesses with a sharp knife. The mango slices were then dipped in various solutions for 5 min. according to the treatment plan (Table 1). After removing from the solution, slices were spread over the perforated tray and well-drained before drying. The control sample was without any pre-treatment. The pre-treated mango slices were subjected to dehydration in a cabinet dryer (Commercial dehydrator systems, Inc., R-5A, USA.) at 60 ± 5 °C. After drying, the dried slices were packed in 25 μm low-density polyethylene bags (150 × 200 mm). During the study, the samples were stored for 180 days in a chamber at 25 ± 2 °C and 70 ± 5% relative humidity.

**Table 1:** Application of pre-treatments on mango slices.

Treatments	Pre-treatments						
	Chemicals			Herbals			
	KMS%	CA%	KMS + CA%	GJ%	LJ%	GJ + LJ%	GJ + LJ%
**T**_**0**_	–	–	–	–	–	–	–
**T**_**1**_	–	1	–	–	–	–	–
**T**_**2**_	1	–	–	–	–	–	–
**T**_**3**_	–	–	1 + 1	–	–	–	–
**T**_**4**_	–	–	–	–	50	–	–
**T**_**5**_	–	–	–	50	–	–	–
**T**_**6**_	–	–	–	–	–	25 + 75	–
**T**_**7**_	–	–	–	–	–	–	75 + 25

T_0_; Control, T_1_; 1% CA, T_2_; 1% KMS, T_3_; 1% KMS + 1% CA, T_4_; 50% LJ, T_5_; 50% GJ, T_6_; 25%GJ + 75%LJ, T_7_; 75%GJ + 25%LJ; Potassium metabisulphite (KMS), Citric acid (CA), Ginger juice (GJ), Lemon juice (LJ)

### Proximate analysis

The proximate compositions of the samples were assessed on a dry basis following standard procedures AOAC [9].

### Physicochemical analysis

#### pH.

The pH of the samples was determined according to the method outlined by AOAC (2012) using a digital pH meter (Spectrum Technologies Inc.). A sample suspension was prepared by homogenizing 20 g of the sample in 100 mL of purified water, after which the pH was measured. Before analysis, the pH meter was calibrated using standard buffer solutions of pH 4.0 and 7.0. The final pH value was expressed as the mean of the recorded measurements.

#### Titratable acidity.

For titratable acidity analysis, 10 mL of fruit juice from each sample was transferred to a beaker and diluted with distilled water at a ratio of 1:4. The titratable acidity was then measured using an acidity meter (GMK-835N, G-WON, Korea) and expressed as citric acid equivalent [10].

#### Total soluble solids.

Total soluble solids (TSS) content was determined using a digital refractometer (HI96801, Hanna Instruments, USA). A drop of fruit juice was applied to the prism surface, and the readings were recorded directly as °Brix at ambient temperature [11].

#### Vitamin C.

Vitamin C (ascorbic acid) content of the mango samples was determined following the method described by Kaushik et al. [10]. For this analysis, 5 mL of the prepared sample was mixed with 95 mL of oxalic acid solution to obtain a final volume of 100 mL. An aliquot of 5 mL from this mixture was then titrated against 2,6-dichlorophenol indophenol dye until a light pink endpoint was observed. The titration readings were recorded, and the mean value was used to calculate the ascorbic acid content. A standard solution containing 1 mL of ascorbic acid and 1.5 mL of oxalic acid was also titrated against the dye to establish the reference value. The vitamin C content was expressed as mg per 100 g of sample.

#### Total sugars.

Total sugar content was determined following the method described by Rashid et al. [12]. Briefly, 50 mL of the prepared sample was heated for 10 min after the addition of 5 g citric acid to facilitate sugar inversion. The sample was then neutralized using sodium hydroxide (NaOH), and the final volume was adjusted to 250 mL with distilled water in a volumetric flask. Total sugar content was subsequently calculated using the appropriate formula:


$$\begin{array}{c} \text{Total sugars }(\%)\;=\;\frac{\text{Factor }\times \text{Dilution }\times \text{1}\text{0}\text{0}}{Weight\;of\;sample\;\times \;Titre} \end{array}$$

### Preparation of sample extract

A 1 g sample was extracted using 10 mL of a methanol–water mixture (60:40, v/v) and agitated at 180 rpm for 30 min at room temperature. The extract was then centrifuged at 2000 rpm for 20 min, after which the resulting supernatant was collected and used for subsequent analyses.

#### Total phenolic content.

Total phenolic content (TPC) of the mango samples was determined using the Folin–Ciocalteu colorimetric method. Briefly, 0.125 mL of the prepared sample extract was mixed with an equal volume of Folin–Ciocalteu reagent and 0.50 mL of distilled water. The mixture was allowed to react for 6 min at room temperature, after which 1.25 mL of 7% sodium carbonate solution was added. The final volume was adjusted to 3 mL with distilled water, and the mixture was incubated for 90 min in the dark. Absorbance was measured at 760 nm using a UV–Vis spectrophotometer (U2020, IRMECO, Germany). The results were expressed as milligrams of gallic acid equivalents per 100 g of sample (mg GAE/100 g) [13].

#### Total flavonoid content.

Total flavonoid content (TFC) of the mango samples was determined following the method described by Abbasi et al. [14], with minor modifications. In brief, 0.25 mL of the prepared sample extract was mixed with 1.25 mL of distilled water, followed by the addition of 0.75 mL of 5% sodium nitrite solution. The mixture was allowed to stand for 6 min at room temperature, after which 0.150 mL of 10% aluminum chloride solution was added. The final volume was adjusted to 2.5 mL with distilled water. Absorbance was measured at 510 nm using a UV–Vis spectrophotometer (U2020, IRMECO, Germany), and the results were expressed as milligrams of catechin equivalents per 100 g of sample (mg CE/100 g).

#### In vitro antioxidant activity.

The antioxidant activity of the mango samples was evaluated using DPPH (2,2-diphenyl-1-picrylhydrazyl) and FRAP (ferric reducing antioxidant power) assays. For the DPPH assay, 0.050 mL of the prepared sample extract was mixed with 2 mL of DPPH reagent, and the absorbance was measured at 515 nm using a UV–Vis spectrophotometer (U2020, IRMECO, Germany). For the FRAP assay, 0.200 mL of the sample extract was combined with 3 mL of FRAP reagent, and the absorbance was similarly recorded at 515 nm. Antioxidant activity results for both assays were expressed as micromoles of Trolox equivalents per gram of sample (μmol TE/g) [10].

### Microbial analysis

The total plate count of the mango samples was determined according to the method of Vithana et al. (2018). First, 0.9% NaCl solution was synthesized by dissolving 0.9 g of sodium chloride in 10 ml of deionized water. A 1:10–6 dilution was precisely produced by adding a uniform sample to the volume of diluent necessary. Nutrient agar contained 2.8 grams of nutrient broth and was made up to 100 mL with deionized water and autoclaved. Finally, 200 µL of the sample was spread aseptically on nutrient agar petri dishes and allowed to stand at 37 ± 0.5°C for 24 h. Colony-forming units (CFU) of bacteria were determined, and the data were expressed as log CFU g–1 [13].

### Color analysis

The sample was positioned on the measurement platform of a colorimeter (Chroma Meter CR-400, Konica Minolta, Inc., Japan), and a beam of light was directed onto it. The color parameters were recorded in terms of *L*, *a*, and *b* values [10].

### Statistical analysis

Every experiment was performed in triplicate, and the best statistical design factorial under a fully randomized design (CRD) was used to analyze the results. Each replication consisted of a single, separately packed bag of dried mango slices. The experiment consisted of 2 factors, one of which was treatments and the other was storage interval. At each storage interval, to avoid contamination or variation in quality during storage, three randomly selected bags from each treatment were opened once and subjected to the analyses. Tukey’s HSD test in Statistix 8.1 software was used to compare means for significant differences [15].

## Results and discussion

### Characterization of proximate composition during storage

This is a typical occurrence in dried fruits, where exposure to ambient air and packing conditions causes moisture migration. According to Tables 2-7 moisture had a highly significant (p < 0.05) impact during storage. For instance, moisture in T_0_ rose by 2.69%, from 14.11% at 0 months to 16.80% at 6 months (Table 8). The reason for these increases is that dried mango slices are hygroscopic, meaning they take in moisture from their surroundings [16]. T_6_ and T_1_ showed the least increases in water content during storage, indicating the protective capacity of pre-treatments to reduce water absorption. Similar increase in moisture values in dried apricots during storage were also reported by Güzel *et al*. [17]. The initial crude protein content varied slightly between 2.10% and 2.11% across all treatments (Table 8). All treatments showed a slow decrease (p < 0.05) in crude protein content over the storage time. The protein content in the control group (T_0_) dropped by about 9%, from 2.11 ± 0.06% at 0 days to 1.92 ± 0.04% at 180 days. Other treatments showed similar patterns, however the degree of reduction differed slightly. There are several reasons why there is a decrease. Amino acid residues break down, and secondary products are formed as a result of protein oxidation, a common phenomenon during storage. The results of the study are consistent with previous research conducted by Bai et al*.* [18]. Over the course of the storage period, T_6_ showed the highest mean protein content (2.065%) among the treatments, whereas T_0_ showed the lowest (2.015%), demonstrating T_6_’s good ability to preserve protein content. Table 8 shows that crude fiber content was greatly impacted by storage time, with values falling from 2.113% at 0 months to 2.058% at 6 months.

**Table 2:** ANOVA for moisture content.

Source	DF	Adj SS	Adj MS	F-Value	P-Value
Treatment	7	79.090	11.2986	23.18	0.000^**^
Storage Days	3	28.162	9.3873	19.26	0.000^**^
Treatment*Storage Days	21	3.610	0.1719	0.35	0.995^NS^
Error	64	31.193	0.4874		
Total	95	142.055			

** = P < 0.01 Highly significant * = P < 0.05 Significant NS = P> 0.05 non-significant.

**Table 7:** ANOVA for NFE.

Source	DF	Adj SS	Adj MS	F-Value	P-Value
Treatment	7	53.164	7.5948	12.83	0.000**
Storage Days	3	35.862	11.9540	20.20	0.000**
Treatment*Storage Days	21	0.086	0.0041	0.01	1.000^NS^
Error	64	37.879	0.5919		
Total	95	126.991			

** = P < 0.01 Highly significant * = P < 0.05 Significant NS = P> 0.05 Non-significant

**Table 8:** Mean values for the proximate composition (%) of dried mango slices during storage interval of 180 days.

Treatments	Storage intervals				Means
	0	60	120	180	
**Moisture**					
**T**_**0**_	14.11 ± 0.78^bcd^	14.77 ± 0.75^abc^	15.73 ± 0.65^ab^	16.80 ± 0.46^a^	**15.35** ^**a**^
**T**_**1**_	12.49 ± 1.17^def^	12.93 ± 1.06^cdef^	13.50 ± 0.95^bcdef^	13.93 ± 0.90^bcde^	**13.21** ^**b**^
**T**_**2**_	12.67 ± 0.71^cdef^	12.93 ± 0.74^cdef^	13.30 ± 0.79^cdef^	13.67 ± 0.76^bcde^	**13.14** ^**b**^
**T**_**3**_	13.12 ± 0.65^cdef^	13.40 ± 0.62^cdef^	13.87 ± 0.57^bcde^	14.33 ± 0.61^bcd^	**13.68** ^**b**^
**T**_**4**_	13.11 ± 0.20^cdef^	13.53 ± 0.21^bcdef^	14.07 ± 0.25^cde^	14.77 ± 0.25^abc^	**13.87** ^**b**^
**T**_**5**_	12.61 ± 0.68^cdef^	13.93 ± 0.64^cdef^	13.37 ± 0.67^efb^	13.83 ± 0.64^bcde^	**13.18** ^**b**^
**T**_**6**_	12.10 ± 0.24^f^	12.70 ± 0.30^ef^	12.10 ± 0.30^def^	12.50 ± 0.30^def^	**11.91** ^**c**^
**T**_**7**_	12.52 ± 0.93^cdef^	12.80 ± 0.92^cdef^	13.20 ± 0.92^cdef^	13.67 ± 0.97^bcde^	**13.04** ^**b**^
**Means**	**12.75** ^**c**^	**13.12** ^**bc**^	**13.64** ^**b**^	**14.18** ^**a**^	
**Crude protein**					
**T**_**0**_	2.10 ± 0.06^a^	2.06 ± 0.06^b^	1.98 ± 0.04^d^	1.92 ± 0.04^e^	**2.01** ^**a**^
**T**_**1**_	2.10 ± 0.04^a^	2.08 ± 0.05^ab^	2.04 ± 0.04^c^	1.99 ± 0.04^d^	**2.05** ^**ab**^
**T**_**2**_	2.10 ± 0.07^a^	2.08 ± 0.07^ab^	2.05 ± 0.07^bc^	2.01 ± 0.08 cd	**2.05** ^**ab**^
**T**_**3**_	2.10 ± 0.09^a^	2.07 ± 0.09^ab^	2.04 ± 0.09^c^	2.00 ± 0.10^d^	**2.05** ^**ab**^
**T**_**4**_	2.10 ± 0.05^a^	2.08 ± 0.05^ab^	2.05 ± 0.05^bc^	2.00 ± 0.06^d^	**2.06** ^**b**^
**T**_**5**_	2.10 ± 0.06^a^	2.07 ± 0.06^ab^	2.04 ± 0.06^c^	1.98 ± 0.05^d^	**2.04** ^**b**^
**T**_**6**_	2.10 ± 0.06^a^	2.09 ± 0.06^a^	2.05 ± 0.07^bc^	2.01 ± 0.08 cd	**2.06** ^**b**^
**T**_**7**_	2.11 ± 0.07^a^	2.08 ± 0.05^ab^	2.05 ± 0.05^bc^	2.00 ± 0.05^d^	**2.06** ^**b**^
**Means**	**2.10** ^**a**^	**2.07** ^**ab**^	**2.03** ^**b**^	**1.98** ^**c**^	
**Crude fiber**					
**T**_**0**_	2.11 ± 0.06^a^	2.09 ± 0.07^a^	2.06 ± 0.07^a^	2.03 ± 0.06^ab^	**2.07** ^**a**^
**T**_**1**_	2.11 ± 0.06^a^	2.09 ± 0.07^a^	2.08 ± 0.07^a^	2.06 ± 0.07^a^	**2.08** ^**a**^
**T**_**2**_	2.11 ± 0.07^a^	2.10 ± 0.07^a^	2.08 ± 0.07^a^	2.05 ± 0.07^ab^	**2.08** ^**a**^
**T**_**3**_	2.11 ± 0.08^a^	2.10 ± 0.08^a^	2.09 ± 0.08^a^	2.07 ± 0.08^a^	**2.09** ^**a**^
**T**_**4**_	2.11 ± 0.05^a^	2.09 ± 0.05^a^	2.07 ± 0.05^ab^	2.04 ± 0.05^b^	**2.08** ^**a**^
**T**_**5**_	2.11 ± 0.05^a^	2.10 ± 0.05^a^	2.09 ± 0.05^a^	2.06 ± 0.06^a^	**2.09** ^**a**^
T_6_	2.11 ± 0.03^a^	2.11 ± 0.03^a^	2.09 ± 0.03^ab^	2.08 ± 0.03^b^	**2.09** ^**a**^
**T**_**7**_	2.11 ± 0.04^a^	2.10 ± 0.05^a^	2.09 ± 0.05^a^	2.07 ± 0.05^ab^	**2.09** ^**a**^
**Means**	**2.11** ^**a**^	**2.09** ^**ab**^	**2.08** ^**ab**^	**2.05** ^**b**^	
**Crude fat**					
**T**_**0**_	1.11 ± 0.07^a^	1.09 ± 0.06^ab^	1.06 ± 0.06^b^	1.03 ± 0.05^b^	**1.09** ^**a**^
**T**_**1**_	1.13 ± 0.06^a^	1.12 ± 0.05^a^	1.10 ± 0.05^a^	1.09 ± 0.04^a^	**1.11** ^**a**^
**T**_**2**_	1.13 ± 0.06^a^	1.11 ± 0.05^ab^	1.10 ± 0.05^b^	1.08 ± 0.05^b^	**1.10** ^**a**^
**T**_**3**_	1.13 ± 0.06^a^	1.12 ± 0.06^a^	1.11 ± 0.06^a^	1.09 ± 0.07^a^	**1.11** ^**a**^
**T**_**4**_	1.14 ± 0.07^a^	1.13 ± 0.07^a^	1.11 ± 0.07^ab^	1.08 ± 0.06^b^	**1.11** ^**a**^
**T**_**5**_	1.14 ± 0.06^a^	1.12 ± 0.06^a^	1.10 ± 0.06^ab^	1.08 ± 0.05^b^	**1.10** ^**a**^
**T**_**6**_	1.12 ± 0.04^a^	1.10 ± 0.04^ab^	1.09 ± 0.04^b^	1.07 ± 0.04^b^	**1.09** ^**a**^
**T**_**7**_	1.13 ± 0.05^a^	1.12 ± 0.04^a^	1.11 ± 0.05^a^	1.09 ± 0.05^ab^	**1.11** ^**a**^
**Means**	**1.12** ^**a**^	**1.11** ^**ab**^	**1.09** ^**ab**^	**1.070** ^**b**^	
**Ash**					
**T**_**0**_	2.22 ± 0.09^a^	2.19 ± 0.08^a^	2.15 ± 0.07^a^	2.12 ± 0.07^a^	**2.17** ^**a**^
**T**_**1**_	2.22 ± 0.07^a^	2.20 ± 0.07^a^	2.17 ± 0.06^a^	2.14 ± 0.06^a^	**2.18** ^**a**^
**T**_**2**_	2.22 ± 0.07^a^	2.19 ± 0.06^a^	2.17 ± 0.06^a^	2.14 ± 0.05^a^	**2.18** ^**a**^
**T**_**3**_	2.22 ± 0.06^a^	2.19 ± 0.06^a^	2.16 ± 0.06^a^	2.13 ± 0.06^a^	**2.17** ^**a**^
**T**_**4**_	2.23 ± 0.06^a^	2.21 ± 0.06^a^	2.18 ± 0.06^a^	2.15 ± 0.06^a^	**2.19** ^**a**^
**T**_**5**_	2.23 ± 0.04^a^	2.21 ± 0.04^a^	2.18 ± 0.03^a^	2.15 ± 0.03^a^	**2.19** ^**a**^
**T**_**6**_	2.23 ± 0.05^a^	2.20 ± 0.05^a^	2.17 ± 0.05^a^	2.14 ± 0.05^a^	**2.18** ^**a**^
**T**_**7**_	2.22 ± 0.07^a^	2.20 ± 0.06^a^	2.17 ± 0.06^a^	2.15 ± 0.05^a^	**2.18** ^**a**^
**Means**	**2.22** ^**a**^	**2.19** ^**ab**^	**2.16** ^**bc**^	**2.14** ^**c**^	
**NFE**					
**T**_**0**_	78.33 ± 0.9^bcdef^	77.73 ± 0.87^cdef^	77.16 ± 0.86^ef^	76.60 ± 0.84^f^	**77.45** ^**c**^
**T**_**1**_	79.93 ± 1.18^abc^	79.36 ± 1.16^abcde^	78.80 ± 1.14^abcde^	78.23 ± 1.12^bcdef^	**79.08** ^**b**^
**T**_**2**_	79.75 ± 0.67^abcd^	79.21 ± 0.66^abcde^	78.69 ± 0.66^abcdf^	78.16 ± 0.65^bcdef^	**78.95** ^**b**^
**T**_**3**_	78.97 ± 0.80^abcde^	78.45 ± 0.79^bcdef^	77.93 ± 0.79^cdef^	77.42 ± 0.79^def^	**78.19** ^**bc**^
**T**_**4**_	79.29 ± 0.22^abcde^	78.80 ± 0.22^abcde^	78.29 ± 0.22^bcdef^	77.79 ± 0.22^cdef^	**78.54** ^**b**^
**T**_**5**_	79.79 ± 0.74^abcd^	79.26 ± 0.73^abcde^	78.73 ± 0.73^abcde^	78.20 ± 0.73^bcdef^	**78.99** ^**b**^
**T**_**6**_	81.08 ± 0.24^a^	80.50 ± 0.24^ab^	79.92 ± 0.24^abc^	79.35 ± 0.24^abcde^	**80.21** ^**a**^
**T**_**7**_	79.89 ± 0.98^abcd^	79.32 ± 0.97^abcde^	78.75 ± 0.97^abcef^	78.19 ± 0.96^bcdef^	**79.03** ^**b**^
**Means**	**79.63** ^**a**^	**79.07** ^**ab**^	**78.53** ^**bc**^	**77.99** ^**c**^	

T_0_; Control, T_1_; 1% CA, T_2_; 1% KMS, T_3_; 1% KMS + 1% CA, T_4_; 50% LJ, T_5_; 50% GJ, T_6_; 25%GJ + 75%LJ, T_7_; 75%GJ + 25%LJ; Potassium metabisulphite (KMS), Citric acid (CA), Ginger juice (GJ), Lemon juice (LJ); Means±SD followed by different superscript letters within the table are significantly different.

Enzymatic and oxidative activities that occur during storage are responsible for the reduction, which causes complex carbohydrates to partially break down into simpler components. Fiber content may change during storage as a result of factors like temperature or packing, according to a prior study by Sharma et al*.* [19]. Table 8 illustrates how the crude fat content gradually dropped over the course of the 180-day storage period, from an average of 1.12% at the beginning to 1.07% at the end. The mango slices’ mean crude fat content dropped steadily over time, from an average of 1.12 at the start of the storage period to 1.09 at the end. Both chemical and herbal pre-treatments seemed to have a stabilizing effect on the retention of crude fat, despite the lack of highly significant (p < 0.05) effects. Ash content, which indicates the overall mineral content of the mango slices, similarly dropped from 2.23% at the beginning of storage to 2.14% at the conclusion of 180 days in Yakta, according to the mean values in Table 8. According to Wang et al*.*, fat and ash contents decrease during storage [20], which is consistent with the current observations for all experiments.

Table 8’s mean values reveal that, in the Yakta variety, treatment T_6_ had the highest value at 0 days, while T_0_ had the lowest value at 81.0% and 78.33%, respectively. By the conclusion of storage, these numbers had dropped to 79.35% and 76.60%, respectively. When compared to the untreated control (T_0_), chemical pre-treatments such asT_1_ and T_2_ were successful in maintaining the NFE concentration. Compared to other herbal pre-treatments, the combination of 25% ginger juice and 75% lemon juice (T_6_) performed better in maintaining NFE content. The findings of Alemayehu and Mekonnen, who recorded comparable trends in NFE content during fruit storage research [21], are consistent with the observations made throughout the investigation.

**Table 3:** ANOVA for crude protein content.

Source	DF	Adj SS	Adj MS	F-Value	P-Value
Treatment	7	0.02040	0.002914	0.75	0.630^NS^
Storage Days	3	0.18241	0.060804	15.68	0.000^**^
Treatment*Storage Days	21	0.01255	0.000598	0.15	1.000^NS^
Error	64	0.24813	0.003877		
Total	95	0.46350			

** = P < 0.01 Highly significant * = P < 0.05 Significant NS = P> 0.05 Non-significant.

**Table 4:** ANOVA for crude fiber content.

Source	DF	Adj SS	Adj MS	F-Value	P-Value
Treatment	7	0.005607	0.000801	0.23	0.978^NS^
Storage Days	3	0.040103	0.013368	3.78	0.015*
Treatment*Storage Days	21	0.002839	0.000135	0.04	1.000^NS^
Error	64	0.226400	0.003538		
Total	95	0.274949			

** = P < 0.01 Highly significant * = P < 0.05 Significant NS = P> 0.05 Non-significant.

**Table 5:** ANOVA for crude fat content.

Source	DF	Adj SS	Adj MS	F-Value	P-Value
Treatment	7	0.014962	0.002137	0.75	0.629^NS^
Storage Days	3	0.038321	0.012774	4.49	0.006*
Treatment*Storage Days	21	0.002612	0.000124	0.04	1.000^NS^
Error	64	0.182000	0.002844		
Total	95	0.237896			

** = P < 0.01 Highly significant * = P < 0.05 Significant NS = P> 0.05 Non-significant.

**Table 6:** ANOVA for ash content.

Source	DF	Adj SS	Adj MS	F-Value	P-Value
Treatment	7	0.004083	0.000583	0.17	0.990^NS^
Storage Days	3	0.101508	0.033836	9.93	0.010*
Treatment*Storage Days	21	0.001208	0.000058	0.02	1.000^NS^
Error	64	0.218133	0.003408		
Total	95	0.324933			

** = P < 0.01 Highly significant * = P < 0.05 Significant NS = P> 0.05 Non-significant.

### Physicochemical analysis

Statistically, pH showed a highly significant (p < 0.05) impact during storage the study (Tables 9, 10). At the beginning of storage, the mean pH values (Table 14) varied from 3.61 ± 0.04 (T_1_) to 3.83 ± 0.09 (T_0_). Over the course of 80 days, a progressive decrease was observed in all samples. The mean values toward the end of storage varied between 3.71 and 3.66. To a lesser degree, the untreated control group (T_0_) had the highest mean pH value. Similar pH decreases in dried fruit products were also documented by Mir et al. during storage, and they attributed this decrease to the production of weak organic acids as a result of enzymatic activity [22]. According to the study, pre-treatment strategies significantly alter the pH of dried mango slices, making citric acid and lemon juice useful natural pH reducers that increase product shelf life. The pre-treatment selection had an impact on the pH change associated with storage duration across all treatment groups. By preserving pH stability and offering antioxidative defence against spoiling, the ginger and lemon juice preservation combo (T_6_) proved successful natural preservation. For all treatments, titratable acidity increased marginally (Table 14) from 1.90 at 0 days to 1.93 at the conclusion of the storage period. In terms of preserving the acidity value after storage, T_2_, T_4_, and T_5_ performed comparatively better. The increasing concentration of organic acids is caused by both minor microbial actions and residual enzymatic activities. Weak acids, such as lactic acid, citric acid, and acetic acid, are produced by trace biological reactions under low-moisture storage conditions. These results are consistent with the findings of Kowalska et al. [23], who noted a rising trend in acidity during storage.

**Table 10:** ANOVA for titratable acidity.

Source	DF	Adj SS	Adj MS	F-Value	P-Value
Treatment	7	0.125541	0.017934	5.72	0.000**
Storage Days	3	0.017053	0.005684	1.81	0.154^NS^
Treatment*Storage Days	21	0.009489	0.000452	0.14	1.000^NS^
Error	64	0.200667	0.003135		
Total	95	0.352749			

** = P < 0.01 Highly significant * = P < 0.05 Significant NS = P> 0.05 Non-significant.

**Table 9:** ANOVA for pH.

Source	DF	Adj SS	Adj MS	F-Value	P-Value
Treatment	7	0.28694	0.040992	11.74	0.000**
Storage Days	3	0.02879	0.009595	2.75	0.050*
Treatment*Storage Days	21	0.07562	0.003601	1.03	0.441^NS^
Error	64	0.22340	0.003491		
Total	95	0.61475			

** = P < 0.01 Highly significant * = P < 0.05 Significant NS = P> 0.05 Non-significant.

TSS levels varied from 65.35 ± 0.09 (T_3_) to 65.67 ± 0.23 (T_6_) at the beginning of the storage study, as shown in Table 14. The concentration of sugars and other soluble solids during the drying process is influenced by the variations in pre-treatments. TSS levels increased modestly in all treatments during the first two months of storage, reaching a peak of 66.32 ± 0.24 in T_6,_ showing a strong impact (p < 0.05) during storage (Tables 11-13). This rise is explained by moisture equilibration in the dried mango slices, where bound water redistributes and concentrates soluble substances like organic acids and sugars. As water activity in the fruit matrix stabilizes in the early phases of preservation, these tendencies are frequently seen in dried fruit products. But after the 180 days, a decrease in TSS was noted as storage went on, with values ranging from 64.70 (T_3_) to 65.19 (T_7_). The oxidative breakdown of sugars and the volatilization of specific organic compounds, processes that are known to take place in dry goods over extended storage, can account for this decrease. The work by Alam et al*.* examined the storage effect on dried peaches and found that such a declining trend in TSS values, was linked to the reported results for TSS [24].

**Table 11:** ANOVA for total soluble solids content.

Source	DF	Adj SS	Adj MS	F-Value	P-Value
Treatment	7	1.2866	0.18381	2.99	0.009^NS^
Storage Days	3	22.6797	7.55989	122.91	0.000**
Treatment*Storage Days	21	0.0006	0.00003	0.00	1.000^NS^
Error	64	3.9365	0.06151		
Total	95	27.9034			

** = P < 0.01 Highly significant * = P < 0.05 Significant NS = P> 0.05 Non-significant.

**Table 13:** ANOVA for total sugar content.

Source	DF	Adj SS	Adj MS	F-Value	P-Value
Treatment	7	0.00785	0.00112	0.25	0.970^NS^
Storage Days	3	4.60638	1.53546	344.80	0.000**
Treatment*Storage Days	21	0.00171	0.00008	0.02	1.000^NS^
Error	64	0.28500	0.00445		
Total	95	4.90093			

** = P < 0.01 Highly significant * = P < 0.05 Significant NS = P> 0.05 Non-significant.

The Yakta variety’s initial vitamin C concentration varied between 57.63 ± 0.11 mg/100g for T_0_ and 62.12 ± 0.10 mg/100g for T_4_, according to the Table 9 Because there were none to protect against oxidative destruction, the untreated control (T_0_) had the lowest initial vitamin C concentration. On the other hand, because of the high ascorbic acid content of lemon juice, which directly adds to the overall vitamin C content and offers a potent antioxidative shield, T_4_ maintained the greatest levels (p < 0.05) of vitamin C at the beginning of Yakta. According to earlier research by Hossain et al. fruit products’ ascorbic acid concentration can be successfully increased by natural acidulants like lemon juice, which serve as a preservative and a nutrient [25]. All treatments demonstrated a consistent decrease in vitamin C concentration over storage, with mean values falling from 59.99 mg/100g at 0 days to 50.47 ± 0.10 mg/100g at 180 days. Because ascorbic acid is thermally and oxidatively unstable, this decrease is anticipated. According to Badin et al. [26], extended exposure to light, oxygen, and residual moisture speeds up the breakdown of vitamin C. From 57.63 ± 0.11 mg/100g at the beginning to 47.05 ± 0.10 mg/100g at the end of storage days, the untreated control (T_0_) showed the sharpest fall. This illustrates how unprotected slices are susceptible to environmental elements while being stored.

An average of According to Table 14, the total sugar content of the control treatment (T_0_) decreased slightly but steadily during the course of 180 days of storage, from 47.13 ± 0.05 mg/100g to 46.53 ± 0.08 mg/100g. All pre-treated samples showed similar tendencies, with little difference between treatments. For example, T_7_ fell from 47.13 ± 0.06 mg/100g to 46.54 ± 0.09 mg/100g, while T_2_ decreased from 47.14 ± 0.06 mg/100g to 46.56 ± 0.09 mg/100g. The stability of sugars in dried mango slices is highlighted by this constancy in initial levels, regardless of the pre-treatment used. Research has confirmed that antioxidant treatments have little effect on the sugars in dried fruits since their primary breakdown pathway is connected to chemical events during storage rather than enzymatic activity [27]. However, a significant factor in the noted decrease in total sugars was the length of storage. Non-enzymatic browning events, such as the Maillard reaction, which happen over extended storage and include reducing sugars reacting with amino acids, are responsible for the slow decline.

**Table 14:** Mean values for the Physicochemical analysis of dried mango slices during storage interval of 180 days.

Treatments	Storage Intervals				Means
	0	60	120	180	
**pH value**					
**T**_**0**_	3.83 ± 0.09^a^	3.81 ± 0.09^ab^	3.78 ± 0.09^abc^	3.71 ± 0.06^abcd^	**3.78** ^**a**^
**T**_**1**_	3.61 ± 0.04^cd^	3.60 ± 0.04^cd^	3.58 ± 0.03^d^	3.71 ± 0.15^abcd^	**3.62** ^**c**^
**T**_**2**_	3.68 ± 0.02^abcd^	3.65 ± 0.02^abcd^	3.64 ± 0.02^abcd^	3.60 ± 0.03^cd^	**3.64** ^**bc**^
**T**_**3**_	3.64 ± 0.03^abcd^	3.63 ± 0.02^bcd^	3.60 ± 0.02^cd^	3.61 ± 0.03^cd^	**3.62** ^**c**^
**T**_**4**_	3.70 ± 0.07^abcd^	3.68 ± 0.06^abcd^	3.66 ± 0.06^abcd^	3.58 ± 0.02^d^	**3.65** ^**bc**^
**T**_**5**_	3.75 ± 0.07^abcd^	3.73 ± 0.07^abcd^	3.74 ± 0.06^abcd^	3.71 ± 0.07^abcd^	**3.73** ^**a**^
**T**_**6**_	3.74 ± 0.09^abcd^	3.71 ± 0.08^abcd^	3.70 ± 0.07^abcd^	3.72 ± 0.04^abcd^	**3.71** ^**ab**^
**T**_**7**_	3.72 ± 0.05^abcd^	3.73 ± 0.04^abcd^	3.72 ± 0.03^abcd^	3.67 ± 0.03^abcd^	**3.71** ^**ab**^
**Means**	**3.71** ^**a**^	**3.69** ^**ab**^	**3.67** ^**ab**^	**3.66** ^**b**^	
**Acidity (%)**					
**T**_**0**_	1.81 ± 0.06^a^	1.84 ± 0.06^a^	1.87 ± 0.06^a^	1.91 ± 0.07^a^	**1.85** ^**c**^
**T**_**1**_	1.96 ± 0.03^a^	1.98 ± 0.04^a^	1.98 ± 0.02^a^	1.99 ± 0.01^a^	**1.97** ^**a**^
**T**_**2**_	1.91 ± 0.07^a^	1.92 ± 0.07^a^	1.93 ± 0.06^a^	1.95 ± 0.06^a^	**1.93** ^**ab**^
**T**_**3**_	1.95 ± 0.04^a^	1.96 ± 0.04^a^	1.96 ± 0.04^a^	1.98 ± 0.04^a^	**1.96** ^**ab**^
**T**_**4**_	1.90 ± 0.08^a^	1.89 ± 0.05^a^	1.89 ± 0.04^a^	1.90 ± 0.04^a^	**1.89** ^**bc**^
**T**_**5**_	1.88 ± 0.05^a^	1.89 ± 0.05^a^	1.90 ± 0.05^a^	1.91 ± 0.05^a^	**1.89** ^**bc**^
**T**_**6**_	1.90 ± 0.06^a^	1.91 ± 0.06^a^	1.92 ± 0.06^a^	1.93 ± 0.06^a^	**1.91** ^**bc**^
**T**_**7**_	1.89 ± 0.07^a^	1.90 ± 0.08^a^	1.91 ± 0.08^a^	1.93 ± 0.07^a^	**1.90** ^**abc**^
**Means**	**1.90** ^**a**^	**1.91** ^**a**^	**1.92** ^**a**^	**1.93** ^**a**^	
**TSS** ^**o**^**B**					
**T**_**0**_	65.65 ± 0.09^f^	66.31 ± 0.09^ab^	65.98 ± 0.09^abcd^	64.99 ± 0.09^efgh^	**65.73** ^**a**^
**T**_**1**_	65.40 ± 0.61^cdefgh^	66.05 ± 0.62^abcd^	65.73 ± 0.62^abcde^	64.75 ± 0.61^gh^	**65.48** ^**a**^
**T**_**2**_	65.62 ± 0.10^f^	66.28 ± 0.10^ab^	65.95 ± 0.10^abcd^	64.96 ± 0.10^efgh^	**65.70** ^**a**^
**T**_**3**_	65.35 ± 0.09^h^	66.00 ± 0.09^abcd^	65.68 ± 0.09^abced^	64.70 ± 0.09^h^	**65.43** ^**a**^
**T**_**4**_	65.51 ± 0.09^g^	66.17 ± 0.09^abc^	65.84 ± 0.09^abcd^	64.85 ± 0.08^fgh^	**65.59** ^**a**^
**T**_**5**_	65.65 ± 0.16^f^	66.31 ± 0.16^ab^	65.98 ± 0.16^abcd^	64.99 ± 0.16^efgh^	**65.73** ^**a**^
**T**_**6**_	65.67 ± 0.23^abcde^	66.32 ± 0.24^a^	66.00 ± 0.23^abcd^	65.01 ± 0.23^efgh^	**65.74** ^**a**^
**T**_**7**_	65.62 ± 0.09^f^	66.28 ± 0.09^ab^	65.95 ± 0.09^abcd^	64.96 ± 0.09^efgh^	**65.70** ^**a**^
**Means**	**65.55** ^**c**^	**66.21** ^**a**^	**65.88** ^**b**^	**64.90** ^**d**^	
**Vitamin C (mg/100g)**					
**T**_**0**_	57.63 ± 0.11^fg^	54.02 ± 0.11^l^	50.59 ± 0.11^q^	47.05 ± 0.10^s^	**52.32** ^**g**^
**T**_**1**_	59.12 ± 0.11^d^	55.91 ± 0.11^h^	52.76 ± 0.11^n^	49.54 ± 0.11^r^	**54.33** ^**e**^
**T**_**2**_	57.81 ± 0.22^ef^	54.19 ± 0.22^kl^	50.76 ± 0.21^q^	47.22 ± 0.21^s^	**52.49** ^**f**^
**T**_**3**_	61.04 ± 0.12^b^	57.83 ± 0.12^ef^	54.64 ± 0.11^j^	51.35 ± 0.11^p^	**56.21** ^**c**^
**T**_**4**_	62.12 ± 0.10^a^	59.21 ± 0.10^d^	56.27 ± 0.10^h^	53.22 ± 0.09^m^	**57.70** ^**a**^
**T**_**5**_	60.31 ± 0.09^c^	57.35 ± 0.09^g^	54.43 ± 0.09^jk^	51.31 ± 0.08^p^	**55.85** ^**d**^
**T**_**6**_	60.94 ± 0.10^b^	58.01 ± 0.10^ef^	55.05 ± 0.09^i^	52.06 ± 0.09^o^	**56.51** ^**b**^
**T**_**7**_	61.00 ± 0.08^b^	58.06 ± 0.08^e^	55.08 ± 0.08^i^	52.09 ± 0.08^o^	**56.55** ^**b**^
**Means**	**59.99** ^**a**^	**56.82** ^**b**^	**53.69** ^**c**^	**50.47** ^**d**^	
**Total sugar content**					
**T**_**0**_	47.13 ± 0.05^a^	46.93 ± 0.06^abc^	46.75 ± 0.08^cdefg^	46.53 ± 0.08^h^	**46.83** ^**a**^
**T**_**1**_	47.14 ± 0.06^a^	46.95 ± 0.07^abc^	46.77 ± 0.10^bcd^	46.55 ± 0.09^efgh^	**46.85** ^**a**^
**T**_**2**_	47.14 ± 0.04^a^	46.96 ± 0.06^ab^	46.78 ± 0.07^ac^	46.56 ± 0.07^defgh^	**46.86** ^**a**^
**T**_**3**_	47.13 ± 0.06^a^	46.94 ± 0.06^abc^	46.75 ± 0.07^bcdefg^	46.54 ± 0.08^gh^	**46.84** ^**a**^
**T**_**4**_	47.14 ± 0.05^a^	46.97 ± 0.06^ab^	46.78 ± 0.07^bc^	46.56 ± 0.08^defgh^	**46.86** ^**a**^
**T**_**5**_	47.13 ± 0.05^a^	46.95 ± 0.06^abc^	46.76 ± 0.08^bcde^	46.54 ± 0.09^gh^	**46.84** ^**a**^
**T**_**6**_	47.13 ± 0.04^a^	46.95 ± 0.06^abc^	46.76 ± 0.06^bcdef^	46.54 ± 0.07^fgh^	**46.84** ^**a**^
**T**_**7**_	47.13 ± 0.06^a^	46.94 ± 0.06^abc^	46.76 ± 0.08^bcd^	46.54 ± 0.09^gh^	**46.84** ^**a**^
**Means**	**47.13** ^**a**^	**46.94** ^**b**^	**46.76** ^**c**^	**46.54** ^**d**^	

T_0_; Control, T_1_; 1% CA, T_2_; 1% KMS, T_3_; 1% KMS + 1% CA, T_4_; 50% LJ, T_5_; 50% GJ, T_6_; 25%GJ + 75%LJ, T_7_; 75%GJ + 25%LJ; Potassium metabisulphite (KMS), Citric acid (CA), Ginger juice (GJ), Lemon juice (LJ); Means±SD followed by different superscript letters within the table are significantly different.

**Table 12:** ANOVA for vitamin C content.

Source	DF	Adj SS	Adj MS	F-Value	P-Value
Treatment	7	331.78	47.397	3333.43	0.000**
Storage Days	3	1204.07	401.356	28227.22	0.000**
Treatment*Storage Days	21	6.13	0.292	20.54	0.000**
Error	64	0.91	0.014		
Total	95	1542.89			

** = P < 0.01 Highly significant * = P < 0.05 Significant NS = P> 0.05 Non-significant.

#### Total phenolic content (TPC).

The effects of several treatments are shown in Table 19. Statistical analysis predicted highly significant (p < 0.05) results (Tables 15-18). T_7_ outperformed the other treatments with the highest mean value (151.46 ± 1.12 mg GAE/100g). Conversely, at the conclusion of the storage period (T_0_), the control showed the lowest mean TPC (106.878 ± 1.44 mg GAE), indicating the susceptibility of untreated slices to oxidative stress and enzymatic degradation, which decreased to 100.19 ± 2.41 mg GAE/100g. Starting at 151.46 ± 1.12 mg GAE at 0 days and holding onto 133.39 ± 1.13 mg GAE at the end of storage day, T_7_ continuously maintained the highest TPC levels across all storage periods when treatment effects were taken into account. This retention rate highlights how well the ginger-lemon mixture stabilizes phenolic chemicals after storage. T_5_ (50% ginger juice) and T_4_ (50% lemon juice) similarly performed well, with TPC levels of 147.53 ± 1.29 mg GAE and 145.38 ± 1.05 mg GAE at 0 days, respectively, which dropped to 127.47 ± 1.38 and 125.23 ± 0.88 after 180 days of storage. Their retention rates, however, were marginally lower than T_7_’s. Similarly, after 180 days of storage, the initial values of T_1_, T_3_, T_2_, and T6 were 131.51 ± 1.13, 128.41 ± 1.72, 127.65 ± 1.49, and 146.55 ± 1.20, respectively. These values were lowered to 113.18 ± 1.12, 110.05 ± 1.72, 109.29 ± 1.49, and 126.49 ± 1.35.

**Table 15:** ANOVA for total phenolic content.

Source	DF	Adj SS	Adj MS	F-Value	P-Value
Treatment	7	12798.5	1828.35	939.20	0.000
Storage Days	3	4486.8	1495.59	768.26	0.000
Treatment*Storage Days	21	56.6	2.70	1.38	0.160
Error	64	124.6	1.95		
Total	95	17466.4			

** = P < 0.01 Highly significant * = P < 0.05 Significant NS = P> 0.05 Non-significant.

**Table 18:** ANOVA for FRAP content.

Source	DF	Adj SS	Adj MS	F-Value	P-Value
Treatment	7	3396.7	485.239	258.19	0.000
Storage Days	3	1846.7	615.576	327.54	0.000
Treatment*Storage Days	21	212.1	10.098	5.37	0.000
Error	64	120.3	1.879		
Total	95	5575.7			

** = P < 0.01 Highly significant * = P < 0.05 Significant NS = P> 0.05 Non-significant.

According to the study, T_7_ is the most successful organic treatment for maintaining TPC in dried mango slices. The bioactive substances found in ginger and lemon, such as gingerol, shogaol, and ascorbic acid, which are known for their strong antioxidant properties, are responsible for this improved retention. Together, these substances neutralise free radicals and prevent phenolic compounds from undergoing oxidative degradation during processing and storage. TPC levels were likewise strongly impacted by storage length, with a steady decrease over time. This time-dependent decrease in TPC is in keeping with studies previously published for dried fruits, where oxidative and enzymatic processes can cause phenolic chemicals to degrade [28].

#### Total flavonoid content (TFC).

Outperforming all other treatments and proving its effectiveness as a natural preservative (p < 0.05), the combination treatment T_7_ had the greatest mean TFC (117.66 ± 0.58 mg QE/100g to 49.63 ± 0.28). The control treatment T_0_, on the other hand, showed the lowest TFC (86.21 ± 1.37 mg QE to 36.37 ± 0.58) and did not entail any pre-treatment. Likewise, treatments T_5_ (116.08 ± 1.11 at 0 days to 48.88 ± 0.55 at 180 days) and T_6_ (113.22 ± 0.54 to 47.71 ± 0.13). The initial values for T_4_, T_3_, T_2_, and T_1_ were 114.96 ± 1.65, 111.57 ± 0.63, 111.52 ± 0.50, and 110.68 ± 0.54. After 180 days of storage, the final values were 48.50 ± 0.69, 47.07 ± 0.27, 47.07 ± 0.20, and 46.68 ± 0.23, respectively. The rate of TFC reduction, however, differed between treatments, indicating that the pre-treatments had varying long-term protective benefits. For example, treatments involving ginger and lemon juice (T_5_, T_6_, and T_7_) showed a reduced deterioration rate, highlighting the significance of these natural antioxidants in extending shelf life, whereas the control (T_0_) showed a dramatic fall in TFC with time. The higher proportion of ginger juice, which contains bioactive substances, may be the cause of the higher total flavonoid content (TFC) found. These substances prevent flavonoids from oxidative deterioration during storage because they have considerable antioxidant activity and function as efficient free-radical scavengers. Antioxidants generated from ginger can also improve the stability of flavonoid molecules and prevent oxidation chain reactions. The citric acid in lemon juice and the phytochemicals in ginger may have worked in combination to further lower oxidation rates, improving flavonoid retention over the course of storage. This pattern is consistent with earlier studies that highlight the significance of antioxidants in maintaining bioactive molecules during post-harvest processing [29].

#### In vitro antioxidant activity.

DPPH scavenging activity varied significantly (p < 0.05) between treatments (Table 19), with T_7_ (91.59 ± 1.18), closely followed by T_5_ (89.88 ± 1.76) and T_4_ (88.68 ± 0.98). In contrast, the control (T_0_) had the lowest mean DPPH activity (76.59 ± 1.56). The initial values for the other treatments, T_6_, T_2_, T_3_, and T_1_, are 87.74 ± 1.22, 85.32 ± 1.64, 84.34 ± 1.31, and 82 ± 1.81, respectively. The storage period significantly influenced DPPH activity, with a steady drop reported over the 180-day storage. This decrease is a result of antioxidants’ unavoidable oxidative deterioration with time, which has been extensively studied in food preservation research. However, treatments like T_7_ and T_5_ revealed slower rates of decline, i.e., exhibiting final values of 81.87 ± 1.18 and 78.02 ± 1.22, respectively, suggesting their superior ability to retain antioxidant activity over extended periods.

**Table 19:** Mean values for TPC, TFC, DPPH, and FRAP of dried mango slices during storage interval of 180 days.

Treatments	Storage intervals				Means
	0	60	120	180	
**TPC**					
**T**_**0**_	113.56 ± 1.44^m^	109.11 ± 1.62^n^	104.65 ± 1.97^o^	100.19 ± 2.41^p^	**106.87** ^**f**^
**T**_**1**_	131.51 ± 1.13^f^	125.40 ± 1.14^h^	119.29 ± 1.13^j^	113.18 ± 1.12^m^	**122.34** ^**d**^
**T**_**2**_	127.65 ± 1.49^g^	121.53 ± 1.49^i^	115.41 ± 1.49^l^	109.29 ± 1.49^n^	**118.46** ^**e**^
**T**_**3**_	128.41 ± 1.72^h^	122.29 ± 1.72^j^	116.17 ± 1.72^k^	110.05 ± 1.72^n^	**119.23** ^**e**^
**T**_**4**_	145.38 ± 1.05^c^	138.66 ± 0.89^d^	131.94 ± 0.89^f^	125.23 ± 0.88^h^	**135.30** ^**c**^
**T**_**5**_	147.53 ± 1.29^b^	140.84 ± 1.32^c^	134.15 ± 1.35^e^	127.47 ± 1.38^g^	**137.49** ^**b**^
**T**_**6**_	146.55 ± 1.20^bc^	139.86 ± 1.25^cd^	133.17 ± 1.30^e^	126.49 ± 1.35^h^	**136.51** ^**c**^
**T**_**7**_	151.46 ± 1.12^a^	145.44 ± 1.12^c^	139.42 ± 1.13^d^	133.39 ± 1.13^e^	**142.42** ^**a**^
**Means**	**136.50** ^**a**^	**130.39** ^**b**^	**124.27** ^**c**^	**118.16** ^**d**^	
**TFC**					
**T**_**0**_	86.21 ± 1.37^c^	64.66 ± 1.02^j^	48.50 ± 0.77^a^	36.37 ± 0.58^o^	**58.93** ^**e**^
**T**_**1**_	110.68 ± 0.54^e^	83.01 ± 0.40^g^	62.24 ± 0.30^k^	46.68 ± 0.23^n^	**75.65** ^**d**^
**T**_**2**_	111.52 ± 0.50^de^	83.64 ± 0.38^g^	62.73 ± 0.29^k^	47.07 ± 0.20^n^	**76.23** ^**d**^
**T**_**3**_	111.57 ± 0.63^de^	83.68 ± 0.47^g^	62.76 ± 0.35^k^	47.07 ± 0.27^n^	**76.27** ^**d**^
**T**_**4**_	114.96 ± 1.65^b^	86.22 ± 1.23^c^	64.67 ± 0.93^j^	48.50 ± 0.69^m^	**78.58** ^**b**^
**T**_**5**_	116.08 ± 1.11^b^	87.06 ± 0.84^c^	65.20 ± 0.76^ij^	48.88 ± 0.55^m^	**79.30** ^**b**^
**T**_**6**_	113.22 ± 0.54^c^	84.92 ± 0.41^f^	63.60 ± 0.18^jk^	47.71 ± 0.13^n^	**77.36** ^**c**^
**T**_**7**_	117.66 ± 0.58^a^	88.25 ± 0.43^b^	66.19 ± 0.33^i^	49.63 ± 0.28^l^	**80.43** ^**a**^
**Means**	**110.23** ^**a**^	**82.68** ^**b**^	**61.98** ^**c**^	**46.48** ^**d**^	
**DPPH**					
**T**_**0**_	76.59 ± 1.56^j^	73.35 ± 1.57^l^	70.10 ± 1.57^n^	66.85 ± 1.57^p^	**71.72** ^**e**^
**T**_**1**_	82.82 ± 1.81^f^	80.24 ± 0.97^h^	77.00 ± 0.97^j^	73.76 ± 0.97^l^	**78.45** ^**d**^
**T**_**2**_	85.32 ± 1.64^d^	80.08 ± 2.71^h^	76.84 ± 2.71^j^	73.60 ± 2.71^l^	**78.95** ^**d**^
**T**_**3**_	84.34 ± 1.31^e^	81.41 ± 1.82^g^	78.17 ± 1.82^i^	74.93 ± 1.82^k^	**79.71** ^**d**^
**T**_**4**_	88.68 ± 0.98^b^	85.47 ± 1.00^d^	82.23 ± 1.00^f^	78.99 ± 1.00^i^	**83.84** ^**bc**^
**T**_**5**_	89.88 ± 1.76^ab^	86.62 ± 1.77^c^	83.38 ± 1.77^e^	80.14 ± 1.77^h^	**85.00** ^**ab**^
T_6_	87.74 ± 1.22^c^	84.50 ± 1.22^e^	81.26 ± 1.22^g^	78.02 ± 1.22^i^	**82.88** ^**c**^
**T**_**7**_	91.59 ± 1.18^a^	88.35 ± 1.18^b^	85.11 ± 1.18^d^	81.87 ± 1.18^f^	**86.73** ^**a**^
**Means**	**85.87** ^**a**^	**82.50** ^**b**^	**79.26** ^**c**^	**76.01** ^**d**^	
**FRAP**					
**T**_**0**_	60.46 ± 0.97^m^	57.50 ± 0.17^n^	54.38 ± 0.33^o^	51.26 ± 0.49^p^	**55.89** ^**f**^
**T**_**1**_	70.57 ± 1.30^h^	67.35 ± 1.32^j^	64.14 ± 1.38^l^	60.96 ± 1.45^m^	**65.75** ^**e**^
**T**_**2**_	73.44 ± 0.96^f^	69.80 ± 1.37^i^	66.27 ± 1.62^k^	62.74 ± 1.88^l^	**68.06** ^**d**^
**T**_**3**_	77.35 ± 0.88^b^	77.18 ± 1.67^b^	73.47 ± 2.15^f^	69.76 ± 2.64^i^	**74.44** ^**ab**^
**T**_**4**_	80.43 ± 1.14^a^	69.33 ± 0.15^i^	66.11 ± 0.28^k^	62.89 ± 0.42^l^	**69.69** ^**d**^
**T**_**5**_	78.14 ± 0.99^b^	73.76 ± 1.08^e^	70.26 ± 1.21^h^	66.76 ± 1.38^j^	**72.22** ^**c**^
**T**_**6**_	83.40 ± 1.14^a^	73.23 ± 1.17^ef^	69.83 ± 1.13^hi^	66.43 ± 1.11^jk^	**73.22** ^**bc**^
**T**_**7**_	81.72 ± 0.83^a^	77.18 ± 1.67^b^	73.47 ± 2.15^f^	69.76 ± 2.64^i^	**75.53** ^**a**^
**Means**	**75.68** ^**a**^	**70.66** ^**b**^	**67.24** ^**c**^	**63.82** ^**d**^	

T_0_; Control, T_1_; 1% CA, T_2_; 1% KMS, T_3_; 1% KMS + 1% CA, T_4_; 50% LJ, T_5_; 50% GJ, T_6_; 25%GJ + 75%LJ, T_7_; 75%GJ + 25%LJ; Potassium metabisulphite (KMS), Citric acid (CA), Ginger juice (GJ), Lemon juice (LJ); Means±SD followed by different superscript letters within the table are significantly different.

At the conclusion of storage, the T_6_ value also dropped to 78.02 ± 1.22. Comparatively, T_5_ (80.14 ± 1.77 at day 180) and T_4_ (78.99 ± 1.00 at day 180) also did well, although marginally less successfully than T_7_. Remarkably, the ultimate values for T_2_ and T_3_ after 180 days of storage were 73.60 ± 2.71 and 74.93 ± 1.82, respectively. Overall storage times, the control group T_0_ (66.85 ± 1.57) had the lowest DPPH activity. This outcome is expected as untreated mango slices don’t have any exterior defences against oxidative deterioration. The control group’s comparatively sharp drop in DPPH activity highlights the significance of pre-treatment techniques in preserving antioxidant qualities throughout storage. All treatments showed a decrease in DPPH activity over time, though the rate of decline varied depending on the treatment. T_7_ had the most consistent antioxidant effect throughout storage, which is consistent with reports that bioactive compound mixtures typically have more potent and long-lasting antioxidant activity than single compounds [30].

As shown in Table 19, treatment effects on FRAP values demonstrate a distinct hierarchy (p < 0.05) in the antioxidant potential of the mango slices according to the kind of pre-treatment used. With a mean of 60.46 ± 0.97 to 51.26 ± 0.49, the control group (T_0_) consistently showed the lowest FRAP value, demonstrating the untreated mango slices’ inherent antioxidant capability. T_6_ had a higher value on the first day, 83.40 ± 1.14, which dropped to 66.43 ± 1.11 after storage. After 180 days, T_7_ had the highest FRAP values, ranging from an initial value of 81.72 ± 0.83 to a final value of 69.76 ± 2.64 (Table 19). Likewise, the initial values of T_4_, T_5_, T_3_, T_2_, and T_1_ were 80.43 ± 1.14, 78.14 ± 0.99, 77.35 ± 0.88, 73.44 ± 0.96, and 70.57 ± 1.30, respectively. However, after 180 days of storage, the levels of T_3_, T_5_, T_4_, T_2_, and T_1_ decreased to 69.76 ± 2.64, 66.76 ± 1.38, 62.89 ± 0.42, 62.74 ± 1.88, and 60.96 ± 1.45, respectively. T_7_’s strong antioxidant stability was demonstrated by its consistently higher FRAP values during all storage intervals. These results are consistent with those of previous studies by Cai et al. [31]. The combined effects of ginger juice and lemon juice may be the cause of the increased DPPH and FRAP levels observed. Lemon gives ascorbic acid and organic acids that improve antioxidant stability, whereas ginger contributes strong antioxidant agents such as gingerols, shogaols, and zingerone. By enhancing free-radical scavenging activity and reducing power, these bioactive components work in an integrated manner to maintain the antioxidant capacity of dried mango slices throughout the storage period.

**Table 16:** ANOVA for total flavonoid content.

Source	DF	Adj SS	Adj MS	F-Value	P-Value
Treatment	7	3925.9	560.8	1169.26	0.000
Storage Days	3	54778.5	18259.5	38068.05	0.000
Treatment*Storage Days	21	396.9	18.9	39.40	0.000
Error	64	30.7	0.5		
Total	95	59131.9			

** = P < 0.01 Highly significant * = P < 0.05 Significant NS = P> 0.05 Non-significant.

**Table 17:** ANOVA for DPPH content.

Source	DF	Adj SS	Adj MS	F-Value	P-Value
Treatment	7	1905.54	272.220	108.56	0.000
Storage Days	3	1290.60	430.200	171.56	0.000
Treatment*Storage Days	21	9.91	0.472	0.19	1.000
Error	64	160.48	2.508		
Total	95	3366.53			

** = P < 0.01 Highly significant * = P < 0.05 Significant NS = P> 0.05 Non-significant.

### Total plate count

Statistical results showed a highly significant (p < 0.05) impact (Table 20). Across all treatments and storage times, the control group (T_0_), which received no pre-treatment, had the highest microbial count (Tables 21-24). The outcome emphasizes how susceptible untreated mango slices are to microbial contamination and development, especially with longer storage times. The untreated samples’ microbial count significantly increased from 3.55 log10 CFU/g at 0 days to 3.60 log10 CFU/g at 180 days, demonstrating the bacteria’s innate propensity to proliferate over time in the absence of effective antibiotic barriers.

**Table 20:** ANOVA for microbial count.

Source	DF	Adj SS	Adj MS	F-Value	P-Value
Treatment	7	13103.4	1871.91	2032.56	0.000**
Storage Days	3	24821.5	8273.83	8983.87	0.000**
Treatment*Storage Days	21	67.1	3.20	3.47	0.000**
Error	64	58.9	0.92		
Total	95	38050.9			

** = P < 0.01 Highly significant * = P < 0.05 Significant NS = P> 0.05 Non-significant.

**Table 21:** Mean values for microbial count (CFU/g) of dried mango slices during storage interval of 180 days.

Treatments	Storage Intervals				Means
	0	60	120	180	
**Microbial count**					
**T**_**0**_	150.23 ± 0.91^k^	160.24 ± 1.12^h^	174.18 ± 0.99^d^	190.28 ± 0.94^a^	**168.73** ^a^
**T**_**1**_	136.51 ± 0.79^p^	145.32 ± 1.12^m^	164.64 ± 0.76^f^	179.32 ± 0.96^c^	**159.49** ^b^
**T**_**2**_	132.63 ± 0.95^r^	142.18 ± 0.94^n^	159.72 ± 1.22^i^	174.36 ± 1.10^d^	**156.44** ^c^
**T**_**3**_	139.23 ± 1.07^o^	150.54 ± 0.71^j^	166.74 ± 0.75^e^	181.48 ± 1.18^b^	**152.22** ^d^
**T**_**4**_	127.47 ± 1.11^s^	137.64 ± 0.88^p^	157.55 ± 0.67^i^	171.63 ± 1.20^e^	**148.57** ^e^
**T**_**5**_	118.63 ± 0.79^u^	129.31 ± 1.06^r^	145.44 ± 0.80^m^	161.25 ± 0.97^h^	**138.65** ^f^
**T**_**6**_	115.52 ± 0.79^v^	126.62 ± 0.91^s^	143.42 ± 0.83^n^	158.51 ± 1.30^a^	**136.01** ^g^
**T**_**7**_	114.42 ± 0.63^w^	124.14 ± 1.09^t^	139.65 ± 0.80^o^	154.43 ± 0.90^j^	**133.15^h^**
**Means**	**129.33** ^d^	**139.50** ^**c**^	**156.42** ^**b**^	**171.41** ^**a**^	

T_0_; Control, T_1_; 1% CA, T_2_; 1% KMS, T_3_; 1% KMS + 1% CA, T_4_; 50% LJ, T_5_; 50% GJ, T_6_; 25%GJ + 75%LJ, T_7_; 75%GJ + 25%LJ; Potassium metabisulphite (KMS), Citric acid (CA), Ginger juice (GJ), Lemon juice (LJ); Means±SD followed by different superscript letters within the table are significantly different.

**Table 24:** ANOVA for b* value.

Source	DF	Adj SS	Adj MS	F-Value	P-Value
Treatment	7	104.860	14.9800	24.30	0.000**
Storage Days	3	220.965	73.6551	119.47	0.000**
Treatment*Storage Days	21	3.138	0.1494	0.24	1.000
Error	64	39.458	0.6165		
Total	95	368.422			

** = P < 0.01 Highly significant * = P < 0.05 Significant NS = P> 0.05 Non-significant.

The effectiveness of both natural and artificial antimicrobial agents in preventing microbial development was demonstrated by the significantly reduced microbial counts in all pre-treated samples as compared to the control. At 0 days, T_3_ had a lower value than the control treatment, followed by T_2_ and T_1_, which had starting values of 2.25, 2.28, and 2.30, respectively. T_7_ was the lowest value among the herbal therapies, followed by T_6_, T_5_, and T_4_ at 2.41, 2.45, 2.48, and 2.45, respectively. Up until 120 days of storage, the microbial count increased less in the treatments other than the control. The microbial count grew after 180 days, although the rise differed by sample, with T_2_ (2.31) and T_3_ (2.28) showing the least increase. During storage, T_4_ (2.55) and T_5_ (2.53) exhibited comparable behaviour. After 180 days of storage, T_6_ displays 2.50 and T_7_ displays a lower value of 2.44. T_7_ had the lowest microbiological count of all the herbal remedies. Bioactive substances found in ginger, including zingerone, gingerols, and shogaols, have antibacterial effects against a variety of spoilage microbes. Citric acid and other organic acids from lemon juice lower the product’s pH and make it less feasible for microorganisms to grow. So, the combined effect of natural treatments proven effective way in controlling microbial population. These findings highlight how well natural antimicrobials work to preserve dried mango slices, especially when combined with lemon and ginger liquids. Overall, the treatments demonstrated that the microbial load was below the ISO 2022 permitted limit of 5 log10 CFU g − 1. The findings and patterns of microbial growth during storage in the current investigation align with those of Mongi et el [32].

### Color analysis

The *L* value of untreated samples significantly decreased to 50.70 ± 0.10, further declining to 46.50 ± 0.10 by the conclusion of a six-month storage period. In contrast, the T3 group exhibited a gradual decrease, with a final value of 52.60 ± 0.10 at the end of the storage period (Table 25). Similarly, other treated groups experienced reductions, with values recorded as 51.77 ± 0.15, 51.43 ± 0.15, 50.47 ± 0.15, and 51.20 ± 0.10 for groups T_1_, T_2_, T_4_, and T_6_, respectively. Notably, the values for T_5_ and T_7_ fell below 50 by the end of the storage period. These results indicate alternately that pre-treated samples retained brighter (higher *L* values) significantly compared to the control, which was untreated, implying the strategy of pre-treatment adopted was efficient in preventing browning and color preservation of the dried mango slices. This observation indicated that the pre-treatments stabilized well the color of the dried mango slices during storage. This is consistent with earlier studies demonstrating that antioxidants and acidulants reduce the rate of enzymatic and oxidative browning in dried fruits and vegetables [27]. The current study did not measure PPO and POD activity. Therefore, the discussion of enzymatic browning inhibition is based on previously published research rather than direct enzyme activity measurements.

**Table 25:** Mean values for L*, a* and b* of dried mango slices during storage interval of 180 days.

Treatments	Storage intervals				Means
	0	60	120	180	
**L** *					
**T**_**0**_	52.80 ± 0.10^j^	50.70 ± 0.10^n^	48.60 ± 0.10^r^	46.50 ± 0.10^u^	**49.65** ^**h**^
**T**_**1**_	55.67 ± 0.19^b^	54.33 ± 0.15^e^	53.00 ± 0.10^i^	51.77 ± 0.15^m^	**53.69** ^**b**^
**T**_**2**_	55.21 ± 0.14^c^	54.13 ± 0.15^f^	52.83 ± 0.15^j^	51.43 ± 0.15^n^	**53.40** ^**c**^
**T**_**3**_	55.77 ± 0.13^a^	54.90 ± 0.10^d^	53.80 ± 0.10^h^	52.60 ± 0.10^j^	**54.26** ^**a**^
**T**_**4**_	54.77 ± 0.15^d^	53.47 ± 0.15^h^	51.97 ± 0.15^l^	50.47 ± 0.15^o^	**52.66** ^**e**^
**T**_**5**_	53.58 ± 0.16^h^	52.37 ± 0.15^l^	50.87 ± 0.15^n^	49.27 ± 0.15^φ^	**51.52** ^**g**^
**T**_**6**_	54.78 ± 0.10^d^	53.70 ± 0.10^g^	52.50 ± 0.10^k^	51.20 ± 0.10^n^	**53.04** ^**d**^
**T**_**7**_	53.82 ± 0.08^g^	52.60 ± 0.10^k^	51.20 ± 0.10^n^	49.70 ± 0.10^p^	**51.83** ^**f**^
**Means**	**54.54** ^**a**^	**53.27** ^**b**^	**51.84** ^**c**^	**50.36** ^**d**^	
**a** *					
**T**_**0**_	12.29 ± 0.07^b^	12.50 ± 0.05^b^	12.75 ± 0.05^a^	13.00 ± 0.05^a^	**12.63** ^**a**^
**T**_**1**_	11.20 ± 0.10^k^	11.30 ± 0.10^j^	11.43 ± 0.08^i^	11.53 ± 0.08^h^	**11.36** ^**e**^
**T**_**2**_	11.34 ± 0.24^j^	11.52 ± 0.23^h^	11.67 ± 0.23^f^	11.83 ± 0.23^e^	**11.58** ^**c**^
**T**_**3**_	11.15 ± 0.04^l^	11.35 ± 0.05^j^	11.50 ± 0.05^h^	11.65 ± 0.05^f^	**11.41** ^**e**^
**T**_**4**_	11.34 ± 0.25^j^	11.45 ± 0.22^i^	11.58 ± 0.19^g^	11.75 ± 0.18^e^	**11.53^cd^**
**T**_**5**_	11.11 ± 0.02^l^	11.26 ± 0.02^k^	11.39 ± 0.04^i^	11.52 ± 0.03^h^	**11.31** ^**e**^
**T**_**6**_	11.16 ± 0.01^l^	11.33 ± 0.03^j^	11.48 ± 0.03^h^	11.63 ± 0.03^f^	**11.40** ^**e**^
**T**_**7**_	11.97 ± 0.06^d^	12.12 ± 0.06^c^	12.27 ± 0.06^b^	12.47 ± 0.06^b^	**12.20** ^**b**^
**Means**	**11.44** ^**d**^	**11.60** ^**c**^	**11.75** ^**b**^	**11.92** ^**a**^	
**b** *					
**T**_**0**_	44.55 ± 1.05^h^	42.67 ± 0.74^l^	41.00 ± 0.70^o^	39.47 ± 0.64^r^	**41.92** ^**c**^
**T**_**1**_	46.88 ± 1.05^b^	45.80 ± 0.95^e^	44.30 ± 0.90^i^	42.83 ± 0.85^l^	**44.95** ^**ab**^
**T**_**2**_	46.61 ± 0.53^c^	45.63 ± 0.47^f^	44.03 ± 0.47^j^	42.37 ± 0.42^m^	**44.66** ^**b**^
**T**_**3**_	47.69 ± 1.05^a^	46.60 ± 0.75^c^	45.23 ± 0.71^g^	43.90 ± 0.75^j^	**45.85** ^**a**^
**T**_**4**_	46.30 ± 0.05^d^	45.50 ± 0.10^f^	44.00 ± 0.10^j^	42.50 ± 0.10^m^	**44.57** ^**b**^
**T**_**5**_	46.13 ± 0.74^d^	45.10 ± 0.82^h^	43.60 ± 0.82^k^	42.23 ± 0.76^n^	**44.26** ^**b**^
T_6_	46.20 ± 0.89^d^	45.37 ± 0.85^g^	44.03 ± 0.80^j^	42.60 ± 0.80^m^	**44.55** ^**b**^
**T**_**7**_	46.27 ± 1.04^d^	45.40 ± 1.10^g^	44.00 ± 1.10^j^	42.60 ± 1.10^m^	**44.56** ^**b**^
**Means**	**46.32** ^**a**^	**45.25** ^**b**^	**43.77** ^**c**^	**42.31** ^**d**^	

T_0_; Control, T_1_; 1% CA, T_2_; 1% KMS, T_3_; 1% KMS + 1% CA, T_4_; 50% LJ, T_5_; 50% GJ, T_6_; 25%GJ + 75%LJ, T_7_; 75%GJ + 25%LJ; Potassium metabisulphite (KMS), Citric acid (CA), Ginger juice (GJ), Lemon juice (LJ); Means±SD followed by different superscript letters within the table are significantly different.

The untreated controls had the highest *a* value of 12.29 ± 0.07 at the beginning of the storage, which gradually increased during storage, showing a highly significant (p < 0.05) impact of treatments (Table 25). The *a* value of T_0_ was 13.00 ± 0.05 at the end of the storage period. Such an increase in the *a* magnitude of the dulled red zone and or redraw zone, possibly due to natural decay of pigment in fruits, and this may be as a result of ongoing oxidative process and or enzymatic activity in the absence of preservative. The changes in the red parameter (*a*) found in control samples might also be related to the Maillard reaction (generating reddish-browner colors) that frequently increases during the storage of food products [33]. In contrast, the pre-treated groups exhibited different extents of decrease of *a*, which revealed a trend toward a less reddish and more neutral (greener) color than that of the untreated control. While the values for chemical treatment T_1_, T_2_, and T_3_ at four months post-stored sample were 11.43 ± 0.08, 11.67 ± 0.23, and 11.50 ± 0.05, were 11.53 ± 0.08, 11.83 ± 0.23 and 11.65 ± 0.05 at the end of storage, respectively (Table 25). Consequently, it was observed that the herbal pre-treated groups exhibited superior performance, with the *a* values of T_4_ and T_5_ remaining stable. At the end of the 6-month storage period, only a slight increase in *a* values was observed from 11.15 ± 0.04 and 11.34 ± 0.25 to 11.65 ± 0.05 and 11.75 ± 0.18, respectively. This implies that herbal pre-treatments have a retardant effect on the red color against the other treatments by preventing oxidative processes and color stability in mango slices due to the antioxidant capacity of ginger and lemon juice [34].

The *b* is a critical value for the investigation of the storage process of dried mango slices because mangoes, which have a characteristic yellow hue, are an indication of better quality and freshness. This parameter is crucial for quality in food processing, that is, the final product meets or even surpasses consumer’s vision for visual appearance and eating experience. Untreated mango slices had the initial *b* value of 44.55 ± 1.05 and significantly (p < 0.05) reduced to 39.47 ± 0.64 during the complete storage period (Table 25). This decrease implies loss of color, i.e., fading of the yellow color, possibly due to a decrease of carotenoids, especially β-carotene, responsible for the yellow of mangoes. The degradation of carotenoids over time is highly likely in oxidative environments, and the exposure of dried mangoes to air, light, and temperature after storage may have increased the chances of this [35]. The results obtained from the control treatment are consistent with those of previous studies, which observed a gradual decrease in yellow color in dried mangoes during storage, attributed to carotenoid degradation [36].

T_1_ and T_2_ groups (chemically treated groups) had the lowest values of 46.88 ± 1.05 and 46.61 ± 0.53 in the initial stage of the induction period, respectively. The former had a slightly better value of 47.69 ± 1.05 at the start of storage due to the synergistic effect of treatments on maintaining the yellow color of the mango slice. After 180 days of storage, *b* values of T_1_, T_2_, and T_3_ were 42.83 ± 0.85, 42.37 ± 0.42, and 43.90 ± 0.75, respectively. This may indicate that citric acid + potassium metabisulphite was especially effective in maintaining the yellow color during storage. This finding is consistent with the study by Chawafambira *et al*., who observed a similar loss of colour intensity in dried mangoes with and without antioxidants during storage [36].

**Table 22:** ANOVA for L* value.

Source	DF	Adj SS	Adj MS	F-Value	P-Value
Treatment	7	182.536	26.0766	1556.72	0.000**
Storage Days	3	234.723	78.2409	4670.81	0.000**
Treatment*Storage Days	21	10.360	0.4933	29.45	0.000**
Error	64	1.072	0.0168		
Total	95	428.691			

** = P < 0.01 Highly significant * = P < 0.05 Significant NS = P> 0.05 Non-significant.

**Table 23:** ANOVA for a* value.

Source	DF	Adj SS	Adj MS	F-Value	P-Value
Treatment	7	19.0970	2.72814	188.59	0.000**
Storage Days	3	3.0277	1.00923	69.77	0.000**
Treatment*Storage Days	21	0.1552	0.00739	0.51	0.956
Error	64	0.9258	0.01447		
Total	95	23.2057			

** = P < 0.01 Highly significant * = P < 0.05 Significant NS = P> 0.05 Non-significant.

## Conclusion

The current research concluded that dried mango slices can be stored under ambient conditions**.** The various treatments applied had different impacts during the study. It was revealed that the combination of lemon and ginger juice treatments yielded comparable results to those of chemical treatments. It was proven that the dried products can be minimally processed using natural resources to achieve some new and nutritious end products, which are nowadays consumers’ preferences.

## Supporting information

S1 Data Study raw data. (XLSX)
